# Addressing Challenges to Enhance Clinical Research in Portugal: Insights from the OncoT3 Expert Group Delphi Study

**DOI:** 10.7759/cureus.73720

**Published:** 2024-11-15

**Authors:** Catarina Resende, Marta Abreu, José Presa Ramos, José Carda, Luís Costa, Fátima Cardoso, Deolinda Pereira, Encarnação Teixeira, Fernanda S Tonin, Filipa Duarte-Ramos

**Affiliations:** 1 Medicine, University of Coimbra, Agencia de Investigação Clínica e Inovação Biomédica (AICIB), Coimbra, PRT; 2 Medical Oncology, AstraZeneca, Lisbon, PRT; 3 Internal Medicine, Hepatology Unit, Centro Hospitalar Trás-os-Montes e Alto Douro, Vila Real, PRT; 4 Hematology, Hospital da Luz, Lisbon, PRT; 5 Medical Oncology, Centro Hospitalar Universitário Lisboa Norte, Lisbon, PRT; 6 Oncology, Breast Unit, Champalimaud Foundation, Lisbon, PRT; 7 Medical Oncology, Instituto Português de Oncologia do Porto, Porto, PRT; 8 Pulmonology, Lung Cancer Unit, Hospital Companhia União Fabril (CUF) Descobertas, Lisbon, PRT; 9 Health and Technology Research Center, Escola Superior de Tecnologia da Saúde de Lisboa (ESTeSL) - Instituto Politécnico de Lisboa (IPL), Lisbon, PRT; 10 Pharmacy, University of Lisbon, Lisbon, PRT

**Keywords:** clinical research, clinical trial, delphi method, portugal, research framework

## Abstract

Introduction

Over the past decades, clinical research has evolved significantly, driven by advances in regulatory frameworks, technological innovations, and methodological approaches. In Portugal, while there has been progress - such as increased regulatory alignment with European standards and the adoption of digital trial management tools - various challenges remain. These may include, among others, limited access to funding, slower patient recruitment rates, and regulatory hurdles that can delay trial approvals. Our goal was to identify key areas for improvement toward the optimization of clinical research practices in the country.

Methods

A modified three-round Delphi study was conducted online (2023-2024) to achieve a nationwide expert consensus. The scientific committee, composed of seven experts, developed 45 initial statements across five topics: dedication time to clinical research, organization of integrated research centers, conditions for implementing clinical trials, the role of institutional authorities, and patient recruitment and referral. A five-point Likert-type scale was used (1 - ‘strongly disagree’, 2 - ‘disagree’, 3 - ‘neither agree nor disagree’, 4 - ‘agree’, and 5 - ‘strongly agree’) to rate each statement. The consensus threshold was established as a percentage of agreement among participants (≥90% in the first round and ≥85% in the second round). The level of consensus achieved by the panel was discussed by the scientific committee during virtual meetings.

Results

Fifty-one experts completed the exercise (86.4% response rate). Consensus was reached on 32 of the 45 initial statements (71.1%) in the first round, with most of them (n = 20, or 62.5%) presenting high concordance rates (>95%). Four new statements were added for the second round, grounded on the feedback from the experts. By the end of the study, consensus was achieved on 45 out of the 49 final statements (91.8%), with the greatest agreement on the organization of integrated research centers and conditions for implementing clinical trials. Three statements regarding patient recruitment and referral, and one statement on the role of institutional authorities to promote clinical research, did not reach consensus, highlighting the need for further dialogue and innovative solutions in these fields.

Conclusion

The insights of this study can inform health organizations, regulatory agencies, and other stakeholders about the barriers and opportunities to improve clinical research in Portugal. By learning from global best practices and tailoring strategies to local contexts, the country can become a more prominent player in the international community.

## Introduction

Over the past few decades, clinical research - a fundamental field for advancing medical science and healthcare outcomes - has changed significantly worldwide, especially in the design and execution of clinical trials [1]. Progress in regulatory frameworks (e.g., streamlining administrative processes), technological innovations (e.g., leveraging real-world data and digital health technologies), and methodological approaches (e.g., replacing large-scale confirmatory trials with adaptive trial designs, conditional approvals, and pragmatic trials) has also contributed to more efficient and effective trials [2]. Collaborative initiatives between academic institutions, industry, and regulatory bodies were also pivotal in driving these advancements, ensuring that clinical research remains robust and responsive for the improvement of scientific and healthcare areas. The goal is to reduce the time and costs associated with approving clinical trials and bringing innovative technologies to patients while maintaining high standards of scientific rigor and ethical integrity [1,2].

In fact, between 2011 and 2021, the absolute number of registered clinical studies grew by 91% worldwide, with increased participation from Southeast Asia and the Western Pacific. These rates are even more significant in the field of advanced therapy medical products (ATMPs) and gene therapies in oncology, with an expected compound annual growth rate of 23% between 2021 and 2026, which represents more than 15% of large drug pipelines. In 2022, over 300 clinical trials focusing on the effect of these new therapies were ongoing in the European Union [3,4]. Yet, countries such as the United States, Germany, Belgium, and Japan remain leaders in clinical research excellence - driven by strong frameworks (e.g., ecosystem of pharmaceutical companies, research institutions, and service providers - including partnerships with Contract Research Organizations), substantial funding, favorable regulatory environment, and advanced infrastructure [3,5,6].

Portugal, by comparison - even with other European countries of similar size - still has room for expansion in this area. Recent efforts have focused on refining practices and frameworks to boost the effectiveness and competitiveness of clinical research across the country. In 2017, the Portuguese Clinical Research Infrastructure Network (PtCRIN) - a national initiative to facilitate partnerships between industry and academia - was launched. Shortly after, in 2018, the Portugal Clinical Trials platform (supported by the Portuguese Association of the Pharmaceutical Industry (Apifarma) and the Agency for Clinical Research and Biomedical Innovation (AICIB)) was created to further support high-quality scientific and technological activities, optimize trials infrastructure, and attract global research [7,8]. These efforts are probably reflected in the fair increase in the number of clinical trial submissions and approvals over the last few years in the country [9]. According to INFARMED, the Portuguese National Authority of Medicines and Health Products, 141 clinical trials were approved in 2018 (from the 159 submitted studies), compared to 152 in 2022 (from the 230 submitted trials) [8,10].

Despite these positive trends and the country's potential to become a key player in the global clinical research landscape, several challenges still need to be addressed. These include the need for increased scientific funding, streamlined regulatory processes, conditions to implement clinical trials and recruit patients, and increased collaboration between academic institutions and industry [5,11]. Building on these insights, this study aimed at identifying the current barriers and key areas for improvement from the perspectives of researchers and healthcare professionals, with the goal of optimizing clinical research practices in Portugal.

## Materials and methods

Study design

This study was designed as a modified three-round Delphi [12,13], to obtain nationwide consensus on the optimization of practices and frameworks, towards further effective and competitive clinical research in Portugal.

Seven experts with different backgrounds (oncology, pulmonology, neurology, clinical hematology, and internal medicine) and large experience in clinical research in the country composed the scientific committee of the study (average H-index = 35), being supported by a research assistance team that was responsible for the co-design, performance, and anonymous data analysis of the Delphi exercise. Research methods were established prior to the beginning of the study, by means of meetings and email correspondence between the scientific committee and the research assistance team.

In the first stage, the scientific committee performed an exhaustive bibliographic search, discussed the most relevant aspects related to the clinical research process in Portugal during a workshop, and finally developed and validated several statements to be included in two sequential rounds of questionnaires (first and second rounds). The questionnaire was addressed to an expert panel, consisting of 59 invited physicians from public and private institutions widely distributed in the country, to capture any regional specificities.

Because the survey was completed anonymously (online) and no personal data were collected, institutional review board approval was not necessary. Consent from the participants, who were allowed to withdraw from the study at any time, was obtained by email. Procedures followed standards for scientific research and were performed according to the Declaration of Helsinki.

Delphi rounds and consensus meeting

The Delphi questionnaire developed by the scientific committee initially included 45 initial statements (items) in Portuguese, divided into five topics: Topic I: Dedication time to clinical research (n = 6 items), Topic II: Organization of integrated research centers (n = 10 items), Topic III: Conditions for implementing a clinical trial (n = 10 items), Topic IV: Role of institutional authorities to promote clinical research in the country (n = 10 items), and Topic V: Optimizing patients' recruitment and referral for clinical trials (n = 9 items).

The questionnaire was shared with the expert panel by e-mail, and participants were asked to express their degree of agreement with each statement using a five-point, ordinal, Likert-type scale (1 - ‘strongly disagree’, 2 - ‘disagree’, 3 - ‘neither agree nor disagree’, 4 - ‘agree’, and 5 - ‘strongly agree’) in each round. Physicians also had the opportunity to add comments to each statement in free-text boxes [12,14]. Relevant comments from experts during the first round were assessed by the scientific committee, as they could lead to statements’ rephrasing, deletion, or addition (e.g., improve text interpretability). During the second round, experts contrasted their previous personal opinions with other participants’ overall degree of agreement and were allowed to reassess their judgment on statements where consensus was not reached. At the end of the exercise, the scientific committee conducted meetings to discuss the final results and propose recommendations regarding clinical research practices in Portugal. The Delphi study ran between July and December 2023.

Data analysis

For analytical purposes, the answers given to the categories ‘strongly agree’ and ‘agree’, or to the categories ‘strongly disagree’ and ‘disagree’, were aggregated into ‘positive consensus’ and ‘negative consensus’, respectively. The consensus threshold (cut-off concordance) was established as a percentage of agreement among experts for each individual statement, equal to or greater than 90% (≥90%) in the first round, and equal to or greater than 85% (≥85%) in the second round. A statement not reaching consensus during the first round was reconsidered in the following round; the remaining items, at the end of the exercise, were considered to have not reached consensus among experts.

The scores and the level of consensus achieved by panelists were used to analyze the group opinion for each item and grounded the discussion of the scientific committee over the assessed topics. Results were summarized in figures and tables. Discrete variables were reported as counts or proportions, and continuous data as median with interquartile ranges (IQRs) [14,15]. The percentage variation of the concordance ratio between rounds (expressed as percentage points (pp)) was used as a convergence indicator [15].

## Results

**Figure 1:**
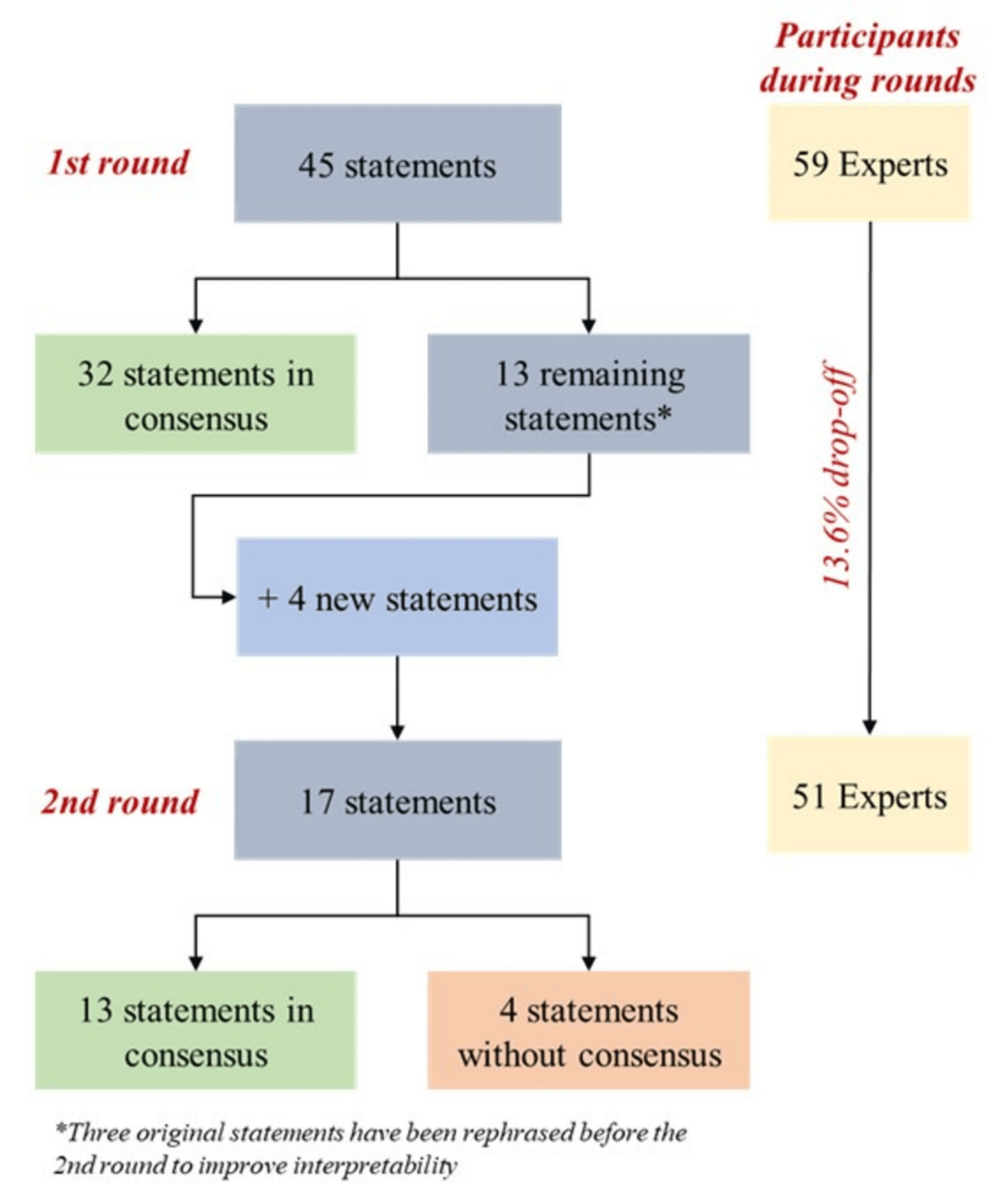
Flowchart of the Delphi exercise

The flowchart of the Delphi exercise is depicted in Figure 1. Overall, 51 of all 59 invited panelists completed all phases of the Delphi exercise (86.4% response rate between rounds). Four new statements (two in Topic I: Dedication time to clinical research; one in Topic IV: Role of institutional authorities to promote clinical research in the country; one in Topic V: Optimizing patients’ recruitment and referral for clinical trials) were developed after the first round by the scientific committee (grounded on panelists' comments) to increase the scope of the study, thus totaling 49 statements assessed during the exercise. The other three statements were reformulated to improve comprehension. The overall results of the two rounds are depicted in Table 1 (in English). For the complete exercise in the original language (Portuguese), see Appendices (Tables 2-3). 

**Table 1:** Summary of findings from the Delphi exercise R1-R4: new statements developed by the scientific committee and added during the second round (grounded on findings from the first round)

No.	Topic	Statement	Round of consensus	Positive agreement	Negative agreement	No. of answers
1	Dedication time to clinical research	No time is dedicated to clinical research	2	98.0%	2.0%	51
2		It is paramount to have time dedicated exclusively to clinical research	1	93.0%	5.3%	57
3		There is no need for allocating time exclusively to clinical research, as is done in basic or translational research	2	5.9%	94.1%	51
4		Clinical research dedication time must be negotiated with hospital administration considering services’ characteristics and needs	2	98.0%	0%	51
R1		Time dedicated to clinical research must be integrated into clinical practice	2	86.3%	9.8%	51
R2		In institutions performing clinical trials, health professionals must have a minimum amount of time in their working schedule dedicated to clinical research	2	98.0%	0%	51
5		Minimal conditions for the implementation of clinical trials, namely flexible consultation time allocated to the trial, should be created	1	100%	0%	57
6		The implementation of clinical trials into practice requires hospital administration investment towards physicians' support, especially considering that consultation within the scope of a trial calls for further time compared to an outside context	1	96.5%	1.8%	57
7	Organization of integrated research centers	Clinical research centers must have management autonomy	1	91.1%	1.8%	56
8		Clinical research centers must have financial autonomy	1	91.1%	1.8%	56
9		Institutions willing to implement clinical trials must have an appropriate professional structure and flow, including a manager responsible for trial timings approval at the board of directors’ level	1	92.9%	1.8%	56
10		Institutions willing to implement clinical trials must have appropriate professional structure and flow, including enough exclusively dedicated coordinators to provide support to the trials	1	96.4%	0%	56
11		Institutions willing to implement clinical trials must have the possibility of hiring members of the research team (e.g., data entry, study coordinators), financed by the clinical research	1	98.2%	1.8%	56
12		It is essential to create working groups between Clinical Research Centers and national authorities aiming at better understanding and discussing the needs for clinical trials promotion in the country (i.e., what needs to be done and how)	1	96.4%	1.8%	56
13		Routine clinical practices of healthcare professionals (physicians, nurses, pharmacists) must integrate clinical research	1	98.2%	0%	57
14		Services of institutions willing to implement clinical trials must have reliable and auditable databases	1	96.5%	0%	57
15		It is paramount to refine service databases (real-time databases) to allow timely informed decision-making	1	94.7%	1.8%	57
16		Services’ databases must be local/regional, yet with the possibility of integrating information from the National Oncological Registry.	2	88.2%	5.9%	51
17	Conditions for implementing a clinical trial	Although there are currently some research centers in Portugal with the appropriate conditions for properly performing clinical research, this is not a standardized scenario for all centers	2	96.1%	0%	51
18		It is essential to reduce the response times of competent institutional authorities to improve the implementation of clinical trials	1	96.5%	1.8%	57
19		According to the Clinical Trials Law, the maximum time recommended for approval of a clinical trial by the regulatory body is 30 days	1	98.2%	0%	57
20		The negotiation of the clinical trial contract with the hospital administration should take place simultaneously with its evaluation aiming at improving trial approval and starting times	1	94.7%	1.8%	57
21		The implementation of studies driven by the researcher (i.e., own initiative) relies on investment in specific resources (e.g., additional human resources or training)	1	98.2%	0%	57
22		There is a need for a professional in charge of managing the trials’ budget	1	91.2%	1.8%	57
23		Minimal conditions for the implementation of clinical trials, namely flexible consultation time allocated to the trial, must be created	1	94.7%	0%	57
24		Minimal conditions for the implementation of clinical trials, namely by means of a coordination unit, must be created	1	94.7%	1.8%	57
25		Minimal conditions for the implementation of clinical trials, namely with enough study coordinators to support trials’ management (including organization of consultations, administrative and logistic tasks), must be created	1	100%	0%	57
26		It is essential to train the structures (services/hospitals) with study coordinators and nurses dedicated to research, enabling their hiring	1	100%	0%	57
27	Role of institutional authorities to promote clinical research in the country	Clinical research through clinical trials should be considered a key factor for Portugal toward its growth, funding, scientific rigor, and international status	1	96.5%	0%	57
28		Hospitals’ investments in clinical trials are quickly recovered	2	90.2%	0%	51
29		To promote clinical research, it is important to define tangible and aligned objectives shared by all stakeholders (inter and intra-institutions, regulators, sponsors), such as doubling the number of clinical trials in 2 years, improving quality metrics, and investing in 2-3 specific and strategic therapeutic areas	1	91.2%	0%	57
30		Hospital funding models considering clinical trial profitability should be developed	2	98.0%	0%	51
31		Hospital administrations are not sufficiently aware of the importance of timely approvals related to clinical research	2	96.1%	0%	51
R3		After the new European regulation, the main barrier to speed-up clinical trial approval is the negotiation process prior to signing the contract by the Board of Directors.	No consensus	72.5%	2.0%	51
32		Hospital administrations are not sufficiently aware of the importance of timely administrative tasks related to clinical research	1	90.9%	3.6%	57
33		Hospital administrations are not sufficiently aware of the profitability of clinical trials	2	90.2%	3.9%	51
34		There is a need to improve response times in the assessment of clinical trials from all national institutional authorities (e.g., CEIC and AICIB)	1	96.5%	0%	57
35		To improve the quality of the evaluation of clinical research, experts working for regulatory authorities must have expertise in the therapeutic area in which a given trial is being proposed	1	94.7%	3.5%	57
36		To increase Portugal's attractiveness for the implementation of clinical trials, decision-makers should recognize the added value of these studies and create an environment to allow timely logistic and administrative processes	1	100%	0%	57
37	Optimizing patients’ recruitment and referral for clinical trials	The size of Portugal is an impediment to the promotion of clinical research at a national level	2	11.8%	86.3%	51
38		Improving patients’ recruitment to clinical trials requires investment in infrastructure	No consensus	80.4%	7.8%	51
39		Improving patients’ recruitment to clinical trials requires investment in organization/management	1	98.3%	0%	57
40		Improving patients’ recruitment to clinical trials requires investment in clinical practice	1	96.6%	1.7%	57
41		A hospital without a specific clinical trial yet that refers patients to another center heading the open study could be entitled to a type of compensation (not necessarily economic) for these participants	No consensus	60.8%	9.8%	51
42		A study coordinator must attend services’ clinical cases meetings/discussions to assist in identifying patients eligible for ongoing clinical trials	No consensus	80.4%	5.9%	51
R4		When discussing clinical cases during multidisciplinary consultations a member of the research team responsible for recruitment (i.e., study coordinator) must be present to assist in identifying eligible patients	2	94.1%	0%	51
43		Clinical trials must integrate routine clinical practice from different services, i.e., the inclusion of a given patient in a study (whenever possible) should be one of the therapeutic options	1	100%	0%	57
44		The development of communication pathways between institutions, aiming at knowing the ongoing clinical trials and their characteristics, is a top priority	1	100%	0%	57
45		The development of patients’ referral models for the ongoing clinical trials is a top priority	1	98.3%	0%	57

During the first round, positive consensus (i.e., due agreement) was reached for 32 of the 45 initial statements (71.1%). Most of these statements (n = 20/32, or 62.5%) had a concordance of over 95% in this round, with one-fifth of them (n = 6 statements) being unanimously agreed upon by participants (rate equal to 100%). Consensual statements in this round were mostly from Topic II: Organization of integrated research centers (n = 9/6 items, or 90.0%) and Topic III: Conditions for implementing a clinical trial (n = 9/6 items, or 90.0%). Statements from the remaining topics (Topic I: Dedication time to clinical research, Topic IV: Role of institutional authorities to promote clinical research in the country, and Topic V: Optimizing patients’ recruitment and referral for clinical trials) were fairly agreed upon (50-60% rates of agreement).

A total of 17 statements (including four newly formulated statements: R1, R2, R3, and R4) were assessed by the panelists in the second round. Almost 80% (n = 13/17) of these statements reached consensus, most of them due to positive agreement (n = 11/13; 84.6%); two items (11.8%) - statements n. 3 (Topic I) and n. 37 (Topic V) - were negatively agreed upon (i.e., consensus due to disagreement). By the end of the Delphi exercise, four statements (8.2%) - one from Topic IV and three from Topic V - did not reach consensus.

Few changes in experts’ responses during the exercise (i.e., between the first and second rounds) were observed, with median variation in agreement and disagreement rates of 8.4% (IQR 4.9; 14.0) and -2.6% (IQR -7.0; 0), respectively. Statements n. 30 (Topic IV), n. 17 (Topic III), and n. 1 (Topic I) presented the highest changes in agreement rates, expressed as 24 pp, 21 pp, and 19 pp, respectively (see Appendices, Table 4).

## Discussion

We were able to perform a nationwide, multidisciplinary Delphi consensus study, whose findings are expected to guide policymakers, researchers, and industry leaders in their efforts to enhance Portugal’s competitiveness and effectiveness in the global clinical research landscape. The high level of compliance among participants from different fields (over 85% between rounds) in our study, combined with ongoing discussions across Europe [16,17] and other regions, such as the U.S., Canada, Australia, and China [18-22], regarding clinical research, underscores the importance of this topic.

Our study was designed as a Delphi exercise - a consensus-based method able to provide a systematic collection and aggregation of informed judgments from a group of experts via multiple iterations. This technique is particularly useful in situations where there is limited empirical data, the problem is complex, and the opinions of experts are critical for further steps or decisions [14]. The controlled feedback from sequential rounds and the anonymity feature of this method support overall consensus, while dominant personality bias is avoided. We also observed a lack of participant attrition throughout the rounds, which increases the validity of the consensus by avoiding the suppression of minority opinions [14,23].

Our study found that around 71% of the initial statements in the questionnaire achieved positive consensus by the end of the first round, with 20% reaching full agreement. Before the launch of the second round, some statements that did not reach consensus during the first round were reformulated by the scientific committee - based on the expert panel's comments, aiming at increasing the clarity of their content. By the end of the Delphi exercise, only four statements (8.2%) were not consensual.

Overall, the statements achieving positive agreement reinforced the increasing role of integrated clinical research units/centers in Portugal, yet there remains a need for these centers to gain organizational and financial autonomy (consensual among over 90% of the panelists: statements 7-16), and for the establishment of standardized conditions for implementing clinical trials (consensual among 95% of experts: statements 17-26). Clinical research centers provide a structured, collaborative environment that supports the research process, including the coordination and management of several aspects of clinical trials (e.g., protocol development, participant recruitment, data collection and analysis, regulatory compliance), provision of infrastructure and resources allocation, and acting as hubs for multidisciplinary networking and partnerships among academic institutions, industries, government agencies, and other research organizations [24]. In this context, it is crucial to centralize clinical trials within these units in Portugal and grant them independence, including the ability to hire staff and access real-time integrated databases (preferably at a national level) to ensure the efficient operation of clinical trials and enable swift decision-making and resource allocation [9,25]. This is consistent with practices in leading research hubs in other countries, where autonomous research centers have contributed significantly to their robust clinical research ecosystems [5,24,26].

Moreover, as clinical trials are increasingly reliant on technological solutions - from big data to artificial intelligence - organizations should strongly invest in adopting new, innovative tools and solutions [16,27]. This is important, as the integrity and reliability of clinical research outcomes rely heavily on access to vast amounts of data. However, the fragmented distribution of this information across multiple institutions, along with ethical and regulatory barriers, may hamper research. In Germany, a medical informatics initiative launched in 2024, with the potential to enable cross-site research on real-world data, may pave the way for intersectoral data sharing and federated analysis in the country [26]. In Belgium, another accessible framework (Federated Learning for Everyone), aiming at facilitating multistakeholder collaboration in clinical research, has recently been introduced [27]. In Portugal, centralized web platforms for clinical studies could be further improved, grounded on these advances from other countries (benchmarking process), by responsible authorities (e.g., INFARMED, CEIC) [7-9].

Despite Portugal's recognized strengths in clinical research in recent years - including high-quality accredited and well-equipped facilities (ranking 27th among 140 economies worldwide) - and qualified clinical research teams, particularly in oncology and neurology [7-9], the conditions for implementing clinical trials remain less than ideal. Our study highlights the critical role of institutional authorities in promoting clinical research, with a particular emphasis on the need for timely approvals and awareness of the profitability of clinical trials (96.1% and 90.2% agreement in the Delphi, respectively). This is a common issue globally - especially with the increasing involvement of non-European countries in trials [28]. Yet, countries with streamlined regulatory processes often experience faster trial initiation and completion times - which is a key factor in selecting certain regions/locations to perform the study. In Europe, Belgium has an average competent authority and ethics committee approval time of 12 days for Phase I trials and 22 days for Phase II, III, and IV trials [5]. In Portugal, the decision on a clinical trial authorization request usually takes around 40-50 days [7,10]. As a response to these challenges, many governments, hospitals, and research centers have developed processes to improve the speed and efficiency of study applications. In a recent systematic review, authors identified around 45 reports of interventions targeting ethics approval processes or governance arrangements, including scope guidelines (to limit ambiguities in the process and fix timelines), streamlined approval (to categorize submissions by risk and triage their review), and mutual recognition (where ethics committees acknowledged other committees' prior reviews) [28].

In addition to robust and efficient regulatory procedures, competent and dedicated clinical trial staff are crucial for ensuring high-quality research. Clinical research coordinators, for instance, play a key role in supporting the clinical research organization, with responsibilities encompassing tasks related to the design, implementation, and evaluation of clinical research trials [29]. Although most panelists agree on the need to dedicate exclusive time to clinical research integrated into clinical practice (as is done in basic and translational research - statement 3), minimal conditions (e.g., flexible consultation times for trials, securing hospital administration investment in physician support) for conducting these activities need to be established (statements R2, 5, and 6). By ensuring that physicians and healthcare professionals have specific time allocated for research activities, institutions can enhance their capacity to conduct rigorous and timely clinical trials [21,28,29].

Our Delphi exercise also highlighted other barriers to implementing clinical trials in Portugal, such as limited patient recruitment and referrals for studies (Topic V), where three statements failed to reach a consensus among experts (statements 38, 41, and 42). In fact, Portugal still has the lowest number of recruited participants per million inhabitants compared to other European countries [8]. This may be due to the ongoing perception of limited involvement of general practitioners in clinical research, the small number of clinical investigators with dedicated research time, and the increasing complexity of regulations and clinical trial contracts in the country - including whether compensations for patients' referral should exist, which may hamper more standardized practices [9,11]. These divergences can also occur due to specific barriers and available resources in a country; while some may focus on cutting-edge infrastructure, others may emphasize organizational efficiency and streamlined processes [20,27,30]. 

Our study has some limitations. The discussion is based on experts' opinions rather than patient data; however, the Delphi method is widely recognized for achieving consensus on a topic in the scientific field. Although many raw statements were included, they reflect the ongoing challenges in clinical research in Portugal. Further statements can be discussed in future studies - particularly those based on non-consensual topics. The Delphi panel included a national group of professionals selected as key opinion leaders; other experts may have slightly different opinions. The findings of this study can ground the development of recommendations to advance clinical research in Portugal.

## Conclusions

In this Delphi exercise, expert consensus statements were generated for the optimization of practices and frameworks for effective and competitive clinical research in Portugal. Several aspects were addressed, including dedication time to clinical research, the organization of integrated research centers, and conditions for implementing a clinical trial. These insights can inform health organizations, regulatory agencies, and other stakeholders on the matter. Areas that did not reach consensus, such as patient recruitment and referral to clinical trials, require further dialogue and innovative solutions. By learning from global best practices and tailoring strategies to local contexts, Portugal can enhance its clinical research landscape, become a more prominent player in the international research community, and bring cutting-edge medicines to patients.

## Appendices

**Table 2:** Overall results (original language: Portuguese) of the first round of the Delphi exercise

Item	Afirmações	Concordo completamente		Concordo		Não concordo nem discordo		Discordo		Discordo completamente		N
		n	%	n	%	n	%	n	%	n	%	
1	Não existe tempo dedicado à investigação clínica (IC).	31	54.4%	14	24.6%	6	10.5%	6	10.5%	0	0.0%	57
2	É crucial a existência de tempo dedicado exclusivamente à investigação clínica.	39	68.4%	14	24.6%	1	1.8%	3	5.3%	0	0.0%	57
3	Não é necessário tempo dedicado exclusivamente à investigação clínica, tal como acontece na investigação básica ou investigação translacional.	2	3.5%	4	7.0%	1	1.8%	17	29.8%	33	57.9%	57
4	O tempo dedicado à IC deve ser negociado com a administração hospitalar, de acordo com as características e necessidades de cada serviço.	23	40.4%	25	43.9%	5	8.8%	3	5.3%	1	1.8%	57
5	Devem ser criadas condições necessárias para a implementação de ensaios clínicos (ECs), designadamente com flexibilidade do tempo de consulta afeta ao EC.	47	82.5%	10	17.5%	0	0.0%	0	0.0%	0	0.0%	57
6	A integração dos ECs na prática clínica exige investimento das administrações hospitalares num verdadeiro apoio aos médicos, desde logo porque uma consulta no âmbito de um EC requer muito mais tempo do que uma consulta fora do contexto do mesmo.	40	70.2%	15	26.3%	1	1.8%	1	1.8%	0	0.0%	57
7	Os centros de investigação clínica têm de ser dotados de autonomia de gestão.	30	53.6%	21	37.5%	4	7.1%	0	0.0%	1	1.8%	56
8	Os centros de investigação clínica têm de ser dotados de autonomia financeira.	33	58.9%	18	32.1%	4	7.1%	0	0.0%	1	1.8%	56
9	Uma instituição que queira implementar ECs tem de ter uma estrutura dedicada, contemplando a existência de um administrador responsável pelos tempos de aprovação dos EC ao nível do conselho de administração.	37	66.1%	15	26.8%	3	5.4%	1	1.8%	0	0.0%	56
10	Uma instituição que queira implementar ECs tem de ter uma estrutura profissionalizada, contemplando existência de coordenadores em número suficiente e em dedicação exclusiva, para prestar um adequado apoio de coordenação dos ECs.	48	85.7%	6	10.7%	2	3.6%	0	0.0%	0	0.0%	56
11	Uma instituição que queira implementar ECs tem de ter a possibilidade de contratação de membros da equipa de investigação (ex: data entry, study coordinators), financiada pela investigação clínica.	45	80.4%	10	17.9%	0	0.0%	1	1.8%	0	0.0%	56
12	É fulcral a criação grupos de trabalho entre os CICs e as autoridades nacionais responsáveis, para conhecimento e debate das reais necessidades para a dinamização de ECs a nível nacional, que possibilitem um melhor conhecimento do que faz falta, do que é preciso fazer e como.	32	57.1%	22	39.3%	1	1.8%	1	1.8%	0	0.0%	56
13	A atividade clínica habitual dos profissionais de saúde (médicos, enfermeiros, farmacêuticos) deve poder integrar a investigação clínica.	40	70.2%	16	28.1%	1	1.8%	0	0.0%	0	0.0%	57
14	O serviço de uma instituição que queira implementar ensaios clínicos tem de ter bases de dados fidedignas e auditáveis.	35	61.4%	20	35.1%	2	3.5%	0	0.0%	0	0.0%	57
15	É necessário profissionalizar as bases de dados dos serviços (real time databases), que permitam a tomada de decisões informadas atempadamente.	38	66.7%	16	28.1%	2	3.5%	1	1.8%	0	0.0%	57
16	As bases de dados dos serviços devem ser locais, mas com a possibilidade de integração da informação constante nas mesmas no Registo Oncológico Nacional.	31	54.4%	19	33.3%	4	7.0%	3	5.3%	0	0.0%	57
17	Em Portugal, atualmente, não existem condições apropriadas para a melhor realização de investigação clínica.	21	36.8%	22	38.6%	10	17.5%	3	5.3%	1	1.8%	57
18	É necessário melhorar os tempos de resposta das autoridades institucionais competentes para melhorar os tempos de implementação dos ECs.	45	78.9%	10	17.5%	1	1.8%	1	1.8%	0	0.0%	57
19	Recomenda-se que o tempo máximo para aprovação de um EC pelas entidades regulamentares seja de 30 dias, de acordo com a lei dos ensaios clínicos.	43	75.4%	13	22.8%	1	1.8%	0	0.0%	0	0.0%	57
20	A discussão do contrato do EC com a administração hospitalar deve decorrer em simultâneo com a avaliação, para melhorar os tempos de aprovação e abertura de ECs.	38	66.7%	16	28.1%	2	3.5%	1	1.8%	0	0.0%	57
21	No que concerne a implementação de estudos da iniciativa do investigador, é necessário o investimento em recursos específicos (recursos humanos adicionais ou capacitação dos recursos existentes).	40	70.2%	16	28.1%	1	1.8%	0	0.0%	0	0.0%	57
22	É necessário a existência de um responsável pela gestão dos orçamentos de forma adequada.	35	61.4%	17	29.8%	4	7.0%	1	1.8%	0	0.0%	57
23	Têm de ser criadas as condições necessárias para a implementação de ensaios clínicos, designadamente com flexibilidade do tempo de consulta afeta ao EC.	42	73.7%	12	21.1%	3	5.3%	0	0.0%	0	0.0%	57
24	Têm de ser criadas as condições necessárias para a implementação de ensaios clínicos, designadamente através da existência de uma unidade de coordenação de EC.	44	77.2%	10	17.5%	2	3.5%	1	1.8%	0	0.0%	57
25	Têm de ser criadas as condições necessárias para a implementação de ensaios clínicos, designadamente através da existência de um número de coordenadores (study coordinators) suficiente para prestar um adequado apoio de coordenação do EC, com a preparação das consultas e de toda a preparação administrativa e logística necessária.	49	86.0%	8	14.0%	0	0.0%	0	0.0%	0	0.0%	57
26	É necessário profissionalizar as estruturas (serviços/hospitais) com coordenadores dos estudos (study coordinators) e enfermeiros dedicados à investigação, com possibilidade de contratação.	46	80.7%	11	19.3%	0	0.0%	0	0.0%	0	0.0%	57
27	A investigação clínica através de ECs deve ser considerada como essencial para Portugal, para o seu crescimento, financiamento, rigor científico e prestígio internacional.	49	86.0%	6	10.5%	2	3.5%	0	0.0%	0	0.0%	57
28	O investimento em ECs num hospital é rapidamente recuperado.	31	54.4%	13	22.8%	13	22.8%	0	0.0%	0	0.0%	57
29	Para promover a IC, é importante a definição de objetivos tangíveis e alinhados e partilhados por todos (inter e intrainstituições, reguladores e sponsors), como por exemplo, duplicar o número de ECs em 2 anos, melhorar as métricas de qualidade, apostar em 2 ou 3 áreas terapêuticas específicas e estratégicas.	24	42.1%	28	49.1%	5	8.8%	0	0.0%	0	0.0%	57
30	Recomenda-se o desenvolvimento de modelos de financiamento hospitalar sustentados na rentabilidade dos ensaios clínicos.	27	47.4%	15	26.3%	11	19.3%	4	7.0%	0	0.0%	57
31	As administrações hospitalares não estão suficientemente sensibilizadas para a importância vital de celeridade e agilidade no que concerne às aprovações relativas à investigação clínica.	29	50.9%	21	36.8%	5	8.8%	2	3.5%	0	0.0%	57
32	As administrações hospitalares não estão sensibilizadas para necessidade de celeridade e agilidade da componente administrativa necessária à investigação clínica.	29	52.7%	21	38.2%	3	5.5%	2	3.6%	0	0.0%	55
33	As administrações hospitalares não estão suficientemente sensibilizadas para a rentabilidade de ensaios clínicos.	24	43.6%	19	34.5%	9	16.4%	3	5.5%	0	0.0%	55
34	É necessário melhorar os tempos de resposta na avaliação de ECs de todas as autoridades institucionais nacionais -- p.ex da CEIC e da AICIB.	39	68.4%	16	28.1%	2	3.5%	0	0.0%	0	0.0%	57
35	Para melhorar a qualidade da avaliação, os peritos que trabalham para as entidades reguladoras devem ter experiência na área terapêutica em que se integram os ECs que avaliam.	36	63.2%	18	31.6%	1	1.8%	2	3.5%	0	0.0%	57
36	Para aumentar a atratividade de Portugal para a implementação de ECs, é fundamental o reconhecimento por parte dos decisores da importância e da mais-valia dos ECs, criando-se condições para agilizar e concretizar os aspetos logísticos e administrativos necessários.	46	80.7%	11	19.3%	0	0.0%	0	0.0%	0	0.0%	57
37	A dimensão de Portugal é um fator impeditivo da promoção da investigação clínica a nível nacional.	3	5.3%	5	8.8%	4	7.0%	23	40.4%	22	38.6%	57
38	A melhoria da capacidade de recrutamento de doentes para ECs requer investimento em infraestruturas.	16	28.1%	26	45.6%	10	17.5%	5	8.8%	0	0.0%	57
39	A melhoria da capacidade de recrutamento de doentes para ensaios clínicos requer investimento em organização.	40	67.8%	18	30.5%	1	1.7%	0	0.0%	0	0.0%	59
40	A melhoria da capacidade de recrutamento de doentes para ensaios clínicos requer investimento na integração dos ECs na prática clínica.	35	59.3%	22	37.3%	1	1.7%	1	1.7%	0	0.0%	59
41	Seria de considerar que um hospital que não tenha um determinado EC e que referencie doentes para esse ensaio aberto noutro centro, pudesse ter direito a uma contrapartida referente a esses doentes.	16	27.1%	17	28.8%	15	25.4%	8	13.6%	3	5.1%	59
42	Na discussão dos casos clínicos do serviço deve estar presente um study coordinator para auxiliar na identificação de doentes elegíveis para os ECs em curso.	25	42.4%	18	30.5%	11	18.6%	5	8.5%	0	0.0%	59
43	Os ECs devem integrar a prática clínica habitual nos diferentes serviços, ou seja, a possibilidade de inclusão de um doente num EC – quando este exista – deve ser uma das opções terapêuticas possíveis.	45	76.3%	14	23.7%	0	0.0%	0	0.0%	0	0.0%	59
44	É urgente criar formas de promover a comunicação entre as instituições, para se saber em tempo real quais os ECs que estão a decorrer e características consideradas relevantes.	47	79.7%	12	20.3%	0	0.0%	0	0.0%	0	0.0%	59
45	É urgente estruturar modelos de referenciação dos doentes para os ECs que estão a decorrer.	43	72.9%	15	25.4%	1	1.7%	0	0.0%	0	0.0%	59

**Table 3:** Overall results (original language: Portuguese) of the second round of the Delphi exercise

Item	Afirmações	Concordo completamente		Concordo		Não concordo nem discordo		Discordo		Discordo completamente		N
		n	%	n	%	n	%	n	%	n	%	
1	Não existe tempo dedicado à investigação clínica (IC).	39	76.5%	11	21.6%	0	0.0%	1	2.0%	0	0.0%	51
3	Não é necessário tempo dedicado exclusivamente à investigação clínica, tal como acontece na investigação básica ou investigação translacional.	0	0.0%	3	5.9%	0	0.0%	4	7.8%	44	86.3%	51
4	O tempo dedicado à IC deve ser negociado com a administração hospitalar, de acordo com as características e necessidades de cada serviço.	26	51.0%	24	47.1%	1	2.0%	0	0.0%	0	0.0%	51
R1	NOVO STATEMENT: O tempo dedicado à IC deve estar integrado na prática clínica.	29	56.9%	15	29.4%	2	3.9%	4	7.8%	1	2.0%	51
R2	NOVO STATEMENT: Na instituições em que decorrem ECs, os profissionais de saúde envolvidos nos mesmos deverão ter contemplado no horário laboral um tempo mínimo dedicado à IC.	40	78.4%	10	19.6%	1	2.0%	0	0.0%	0	0.0%	51
16	As bases de dados dos serviços devem ser locais, mas com a possibilidade de integração da informação constante nas mesmas no Registo Oncológico Nacional.	38	74.5%	7	13.7%	3	5.9%	3	5.9%	0	0.0%	51
17	TEXTO REFORMULADO: Embora, em Portugal, atualmente, já existam alguns centros de investigação com as condições apropriadas para a melhor realização de investigação clínica, em muitos outros tal não se verifica.	40	78.4%	9	17.6%	2	3.9%	0	0.0%	0	0.0%	51
28	O investimento em ECs num hospital é rapidamente recuperado.	39	76.5%	7	13.7%	5	9.8%	0	0.0%	0	0.0%	51
30	TEXTO REFORMULADO: Recomenda-se o desenvolvimento de modelos de financiamento hospitalar que contemplem a rentabilidade dos ensaios clínicos.	41	80.4%	9	17.6%	1	2.0%	0	0.0%	0	0.0%	51
31	As administrações hospitalares não estão suficientemente sensibilizadas para a importância vital de celeridade e agilidade no que concerne às aprovações relativas à investigação clínica.	33	64.7%	16	31.4%	2	3.9%	0	0.0%	0	0.0%	51
R3	NOVO STATEMENT: O principal entrave à celeridade de aprovação dos ECs, após entrada do regulamento europeu, tem que ver com o processo de negociação prévio do contrato financeiro por parte do Conselho de Administração.	17	33.3%	20	39.2%	13	25.5%	1	2.0%	0	0.0%	51
33	As administrações hospitalares não estão suficientemente sensibilizadas para a rentabilidade de ensaios clínicos.	22	43.1%	24	47.1%	3	5.9%	2	3.9%	0	0.0%	51
37	A dimensão de Portugal é um fator impeditivo da promoção da investigação clínica a nível nacional.	1	2.0%	5	9.8%	1	2.0%	25	49.0%	19	37.3%	51
38	A melhoria da capacidade de recrutamento de doentes para ECs requer investimento em infraestruturas.	10	19.6%	31	60.8%	6	11.8%	4	7.8%	0	0.0%	51
41	TEXTO REFORMULADO: Seria de considerar que um hospital que não tenha um determinado EC e que referencie doentes para esse ensaio aberto noutro centro, pudesse ter direito a uma contrapartida, não necessariamente económica, referente a esses doentes.	11	21.6%	20	39.2%	15	29.4%	2	3.9%	3	5.9%	51
42	Na discussão dos casos clínicos do serviço deve estar presente um study coordinator para auxiliar na identificação de doentes elegíveis para os ECs em curso.	25	49.0%	16	31.4%	7	13.7%	3	5.9%	0	0.0%	51
R4	NOVO STATEMENT: No momento de discussão dos casos clínicos do serviço, em consulta multidisciplinar, deve estar presente um elemento da equipa de investigação (incluindo, por exemplo, o study coordinator) dos estudos em recrutamento na instituição, para auxiliar na identificação de doentes elegíveis.	29	56.9%	19	37.3%	3	5.9%	0	0.0%	0	0.0%	51

**Table 4:** Variation of experts’ responses between rounds

Statements	First round - Agreement rates	First round - Disagreement rates	Second round - Agreement rates	Second round - Disagreement rates	Variation - Agreement rates	Variation - Disagreement rates
1	79%	11%	98%	2%	19%	-9%
2	93%	5%	--	--	--	--
3	11%	88%	6%	94%	-5%	6%
4	84%	7%	98%	0%	14%	-7%
R1	--	--	86%	10%	--	--
R2	--	--	98%	0%	--	--
5	100%	0%	--	--	--	--
6	96%	2%	--	--	--	--
7	91%	2%	--	--	--	--
8	91%	2%	--	--	--	--
9	93%	2%	--	--	--	--
10	96%	0%	--	--	--	--
11	98%	2%	--	--	--	--
12	96%	2%	--	--	--	--
13	98%	0%	--	--	--	--
14	96%	0%	--	--	--	--
15	95%	2%	--	--	--	--
16	88%	5%	88%	6%	1%	1%
17	75%	7%	96%	0%	21%	-7%
18	96%	2%	--	--	--	--
19	98%	0%	--	--	--	--
20	95%	2%	--	--	--	--
21	98%	0%	--	--	--	--
22	91%	2%	--	--	--	--
23	95%	0%	--	--	--	--
24	95%	2%	--	--	--	--
25	100%	0%	--	--	--	--
26	100%	0%	--	--	--	--
27	96%	0%	--	--	--	--
28	77%	0%	90%	0%	13%	0%
29	91%	0%	--	--	--	--
30	74%	7%	98%	0%	24%	-7%
31	88%	4%	96%	0%	8%	-4%
R3	--	--	73%	2%	--	--
32	91%	4%	--	--	--	--
33	78%	5%	90%	4%	12%	-2%
34	96%	0%	--	--	--	--
35	95%	4%	--	--	--	--
36	100%	0%	--	--	--	--
37	14%	79%	12%	86%	-2%	7%
38	74%	9%	80%	8%	7%	-1%
39	98%	0%	--	--	--	--
40	97%	2%	--	--	--	--
41	56%	19%	61%	10%	5%	-9%
42	73%	8%	80%	6%	8%	-3%
R4	--	--	94%	0%	--	--
43	100%	0%	--	--	--	--
44	100%	0%	--	--	--	--
45	98%	0%	--	--	--	--
